# How urbanization affects sexual communication

**DOI:** 10.1002/ece3.8328

**Published:** 2021-12-14

**Authors:** Justa L. Heinen‐Kay, Adam D. Kay, Marlene Zuk

**Affiliations:** ^1^ Department of Ecology, Evolution & Behavior University of Minnesota St. Paul USA; ^2^ Biology Department University of St. Thomas St. Paul USA

**Keywords:** city, mating behavior, sexual selection, sexual signal, urbanization

## Abstract

Urbanization is rapidly altering landscapes worldwide, changing environmental conditions, and creating novel selection pressures for many organisms. Local environmental conditions affect the expression and evolution of sexual signals and mating behaviors; changes in such traits have important evolutionary consequences because of their effect on reproduction. In this review, we synthesize research investigating how sexual communication is affected by the environmental changes associated with urbanization—including pollution from noise, light, and heavy metals, habitat fragmentation, impervious surfaces, urban heat islands, and changes in resources and predation. Urbanization often has negative effects on sexual communication through signal masking, altering condition‐dependent signal expression, and weakening female preferences. Though there are documented instances of seemingly adaptive shifts in trait expression, the ultimate impact on fitness is rarely tested. The field of urban evolution is still relatively young, and most work has tested whether differences occur in response to various aspects of urbanization. There is limited information available about whether these responses represent phenotypic plasticity or genetic changes, and the extent to which observed shifts in sexual communication affect reproductive fitness. Our understanding of how sexual selection operates in novel, urbanized environments would be bolstered by more studies that perform common garden studies and reciprocal transplants, and that simultaneously evaluate multiple environmental factors to tease out causal drivers of observed phenotypic shifts. Urbanization provides a unique testing ground for evolutionary biologists to study the interplay between ecology and sexual selection, and we suggest that more researchers take advantage of these natural experiments. Furthermore, understanding how sexual communication and mating systems differ between cities and rural areas can offer insights on how to mitigate negative, and accentuate positive, consequences of urban expansion on the biota, and provide new opportunities to underscore the relevance of evolutionary biology in the Anthropocene.

## Graphical abstract

Urbanization is ubiquitous and alters many environmental features, but we are just beginning to understand the implications for sexual communication in city‐dwelling animals. Our review discusses existing literature on how aspects of urbanization (e.g., noise and light pollution, impervious surfaces, changes in community composition) affect sexual selection and communication.

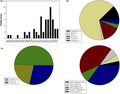

## 1 INTRODUCTION

Eco‐evolutionary research is being performed in a world facing an ever‐intensifying range of environmental and social changes. One crucial driver of this change is urbanization, with over 2.5 billion additional residents predicted to inhabit cities by 2050 (United Nations, 2018). Urbanization affects both biotic and abiotic aspects of the environment, creating novel habitats for the organisms inhabiting cities. Urban environments are characterized by a suite of environmental shifts, including increased ambient noise levels (e.g., from vehicle traffic), artificial light at night, heavy metal pollution, and more impervious surfaces (e.g., buildings, roadways) relative to rural areas less impacted by human activities (Elmqvist et al., 2019; Grimm et al., 2008). Although some features of urban environments may have non‐urban analogs (e.g., artificial light may be similar to constant light at high latitudes), the interaction among these factors almost certainly creates biologically novel selection on many organisms inhabiting urban environments. Organisms in urban areas must either rapidly adapt to the new conditions via evolution or plasticity, or face barriers to urban colonization or population declines and local extinction within urban areas (Alberti et al., 2017; Johnson & Munshi‐South, 2017; Shochat et al., 2006). Though urban evolution is still a young field, a growing body of evidence demonstrates occasional adaptation to this suite of novel conditions, mostly for naturally selected traits like phenology, stress physiology, and cognition (Garroway & Schmidt, 2020; Johnson & Munshi‐South, 2017).

Urbanization can affect traits involved in sexual communication (e.g., coloration, songs, mate choice) that are critical for successful reproduction. While there is a rich body of research investigating how sexual communication is affected by environmental conditions (Cornwallis & Uller, 2010; Gillespie et al., 2014; Henneken et al., 2015; Miller & Svensson, 2014; Servedio & Boughman, 2017), the urban environment requires special consideration (Halfwerk, 2021; Sepp et al., 2020). As environments change due to urbanization, we expect strong selection on both sexual signal expression and receiver responses to these signals for effective communication to occur. Understanding how urbanization shapes sexual communication in animals—both the expression of sexual signals and behavioral responses to them—is important because it directly affects reproduction and fitness. Furthermore, changes in sexual communication can affect evolution within a population regardless of whether trait changes are heritable or reflect phenotypic plasticity (Price et al., 2003). Differences in environmental conditions between populations have often been implicated in causing divergence in sexual signal expression (Cornwallis & Uller, 2010; Martin et al., 2014). Such divergence in sexual traits can cause a breakdown of sexual communication between populations, and lead to reproductive isolation and eventual speciation (Boughman, 2001; Safran et al., 2013; Servedio & Boughman, 2017).

In this review, we focus on the influence of urbanization on sexual communication. While understanding how sexual communication and selection will be affected by a changing planet is beginning to attract more attention (Candolin, 2019; Candolin & Wong, 2012, 2019), work that focuses on urban evolution of sexual traits has received less attention (Sepp et al., 2020). We focus mostly on research that explores how sexual communication is affected by particular environmental aspects of urbanization, but we also draw from work in relatively undisturbed habitats to project how we might expect urban‐associated environmental changes to affect sexual communication. For example, pollution from anthropogenic sources of light, noise, and metals, and the existence of physical structures and impervious surfaces are largely biologically novel, requiring new research approaches to understand their direct effects and their interactions. For other changes like shifts in resource quantity and quality, habitat fragmentation, or increased temperatures, we extrapolate from work that did not explicitly consider urbanization. Our overarching goal is to encourage additional research on how urbanization can affect the sexual selection and evolutionary outcomes in cities, which can, in turn, inspire policies and development that mitigate negative, and promote positive, impacts on the biota.

## 2 BACKGROUND ON SEXUAL COMMUNICATION RELEVANT FOR URBAN ECOLOGY

Elaborate sexual traits often serve as signals that contain information individuals use to make mating decisions. For effective communication to occur, signals first need to be detected by the receiver, and second deemed of sufficient quality. Sexual signals are often costly to produce and maintain, resulting in signals that honestly reflect individual condition (Hill & Montgomerie, 1994; Salvador et al., 1995). Changes in the urban environment may render signals less easily detectable by the receiver, and/or make their information content less reliable than under typical habitat conditions.

Animals often use multiple signals during sexual communication and understanding how and why this occurs is currently a key topic in sexual selection research (Partan, 2013). Different signals, or different components of the same signal, can provide information to receivers that is redundant “backup” information, amplifies information, or offers distinct, complementary information (Candolin, 2003). The existence and use of multiple sexual signals have been hypothesized to help buffer populations from rapid environmental changes because communication would not be completely curtailed when one signal becomes ineffective in novel condition (Partan, 2013). Urbanization may shift the relative efficacy of different signal modalities, providing opportunities for researchers to assess how commonly and under what circumstances adaptive trait compensation may occur. Urban studies might be particularly useful for determining whether signal compensation is more likely to occur when signals convey redundant information about the signaler.

Whether sexual selection hinders or helps adaptation to novel environmental conditions remains an active question in evolutionary biology (Candolin & Heuschele, 2008; Kokko & Brooks, 2003; Servedio & Boughman, 2017). If individuals use highly stringent and inflexible criteria to choose mating partners, then some individuals may forgo reproduction altogether when no available mate is deemed suitable, particularly under poor conditions or when population sizes are small (Kaneshiro, 1976, 1980). However, theory and a growing body of empirical work indicates that sexual selection can facilitate local adaptation, as it operates through differential reproductive success and is directly related to fitness (Dugand et al., 2019; Fricke & Arnqvist, 2007; Holland & Rice, 1999). Sexual selection can increase the speed of local adaptation if natural and sexual selection favor the same traits, and locally adapted individuals contribute more offspring to the next generation (Lorch et al., 2003). By favoring individuals in good condition, sexual selection can also improve population viability by purging deleterious mutations (Jarzebowska & Radwan, 2009; Whitlock & Agrawal, 2009). It is currently unclear how the strength of sexual selection generally changes with urbanization, and whether any changes in sexual selection generally facilitate adaptation to novel urban conditions. It is also important to note that acclimation and adaptation may have less influence than community assembly processes in determining the characteristics of the biota in urban environments. Thus, understanding how sexual communication allows for or prevents colonization of urban areas is also an important area for research.

## 3 LITERATURE REVIEW METHODS

**FIGURE 1:**
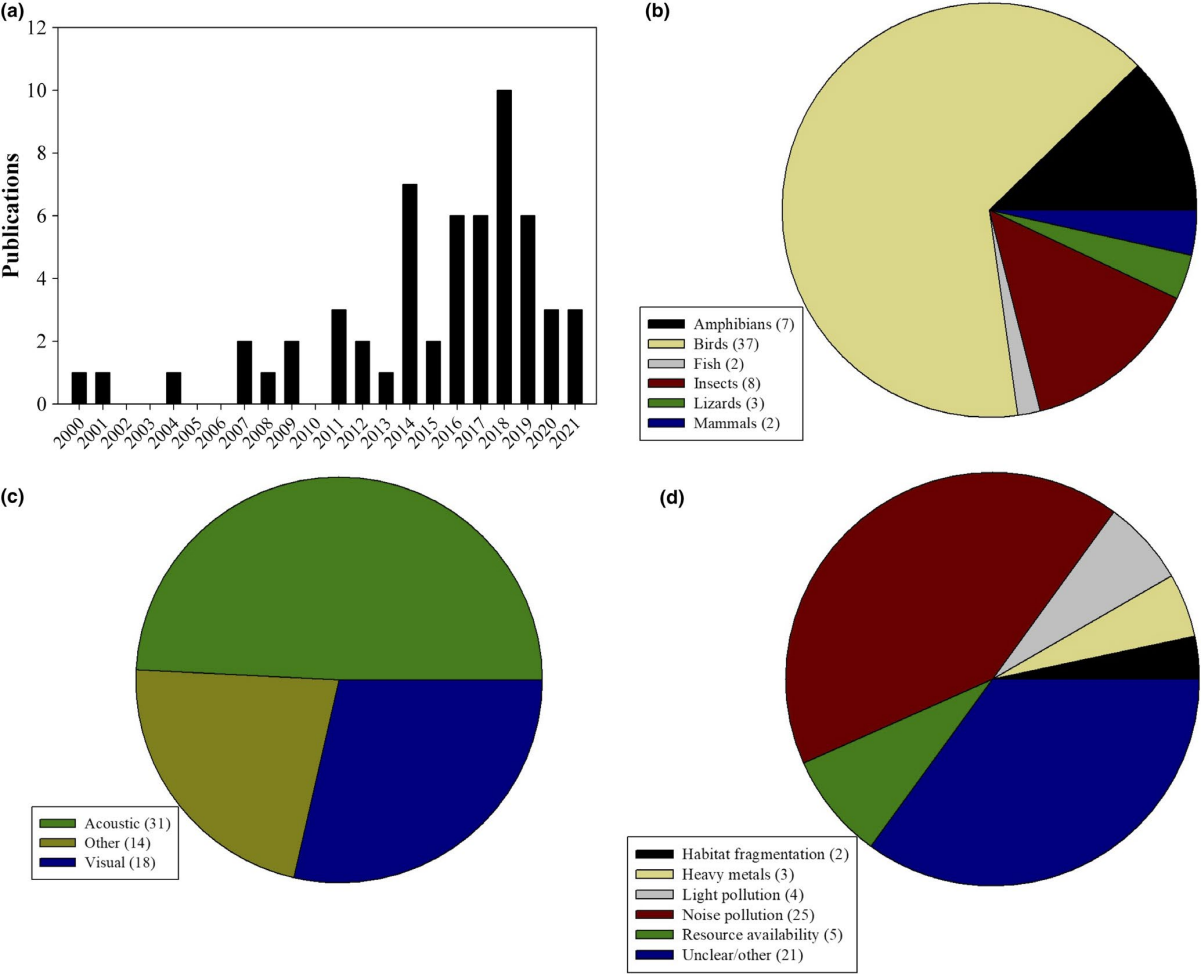
Summary of information from the research papers uncovered by the literature search and included in our table. (a) Distribution of papers by publication year. (b) Focal taxon of the study. (c) Primary signal modality investigated. (d) Aspect of urbanization studied

We conducted a search in ISI Web of Science that included the following search terms, TOPIC: "sexual selection" or "sexual signal*" or "mating behavio*" or "courtship" or "sexual communication" or "mate choice" or "male‐male competition" AND TOPIC: "urban*" or "city" This search was last updated March 3, 2021, and yielded 327 papers in total. We determined the relevance of each paper and excluded reviews, meta‐analyses, empirical studies that did not explicitly examine the city as a selective agent on sexual traits, and studies that broadly investigated consequences of anthropogenic impacts but not urbanization *per se*. For instance, we excluded papers that involved agriculture, consequences of nutrient pollution from fertilizer (e.g., eutrophication, hypoxia), and endocrine‐disrupting chemicals from pharmaceuticals that did not directly address urbanization. We were left with 58 relevant papers from this search that are summarized in Table 1 and Figure 1. Most (75%) of these papers were published since 2014. Most (63%) focused on birds, followed by insects (14%) and amphibians (12%); no studies focused on plants. The aspect of sexual selection that received the most attention in these papers was acoustic signals (54%) followed by visual signals (30%). For the 45 studies that focused on a specific aspect of urbanization, most of these (58%) focused on noise pollution.

**TABLE 1:** Description of studies uncovered in the Web of Science literature search for urbanization and sexual communication (see detailed search terms provided in methods)

Taxa	Species (Common name)	Aspect of sexual selection	Specific Trait	Association with urbanization	Plastic or evolved change	Aspect of urbanization	Reference
*Vertebrates*							
Amphibian	*Bufo raddei*	Acoustic signal Physiology	Song characteristics Physiology	Males produced longer, more complex calls in the polluted site. Larger dermal breeding glands and stronger forearms for amplexus in the polluted area.	Not tested	Metal pollution	Guo et al. (2018)
	*Crinia signifera* (Common eastern froglet) *Litoria ewingii* (Southern brown treefrog)	Acoustic signal	Song characteristics	No difference in call frequency between noisy and quiet sites. Males produce higher frequency calls in noisier areas.	NA Not tested	Noise pollution	Parris et al. (2009)
	*Dendropsophus triangulum* (Amazonian treefrog)	Acoustic signal	Song characteristics	Call rates were higher when paired with traffic noise and music. No difference in call rate when presented with conspecific chorus noise.	Plastic	Noise pollution	Kaiser and Hammers (2009)
	*Litoria ewingii* (brown tree frog)	Acoustic signal	Song characteristics	Traffic noise resulted in a significant short‐term increase in call pitch	Plastic	Noise pollution	HIgham et al. (2021)
	*Hyla arborea* (European treefrog)	Behavior	Mate choice	No difference in the relative importance of visual and acoustic signals during mate choice in presence of traffic noise.	NA	Noise pollution	Troianowski et al. (2014)
	*Hyla chrysoscelis* (Cope's grey treefrog)	Behavior	Mate choice	Females took longer to respond to male calls, oriented toward the playback speaker less, and expressed a higher response threshold when exposed to either traffic noise or conspecific chorus noise.	Plastic	Noise pollution	Bee and Swanson (2007)
	*Physalaemus pustulosus* (Tungara frog)	Acoustic signal Behavior	Song characteristics Mate choice	Urban frogs sing faster, more complex calls than rural frogs. Females from both habitats preferred urban males.	Plastic NA	Urban vs. rural (Noise and light pollution, predation risk)	Halfwerk et al. (2019)
Bird	*Cardinalis cardinalis* (Northern cardinal)	Acoustic signal	Song characteristics	Songs were longer, higher frequency, and faster in urban areas. Higher frequency songs were specifically associated with noise pollution, while rate and song length were most associated with conspecific density. No association between vegetation and song aspects.	Not tested	Urban vs. rural (Noise, vegetation, and conspecific density)	Narango and Rodewald (2016)
		Visual signal	Coloration Territory quality Reproductive success	In rural areas, more colorful males bred earlier and gained higher quality territories, but achieved lower reproductive success (likely due to evolutionary trap with exotic nesting plant). In urban areas, there was no relationship between male coloration, territory quality, and reproductive success.	Not tested	Urban vs. rural (Exotic nesting plant)	Rodewald et al. (2011)
	*Columbia livia* (Pigeon)	Visual signal	Coloration	Lead decreased brightness of iridescent and melanic feather coloration, while zinc increased reflectance of melanic feathers.	Plastic	Metal pollution	Chatelain et al. (2017)
	*Copsychus saularis* (Magpie‐robin)	Acoustic signal	Song characteristics	In urban areas, birds sang longer, slower songs than rural birds. No difference in song complexity.	Not tested	Urban vs. rural	Hill et al. (2018)
	*Dumatella carolinensis* (Gray catbird)	Extra‐pair paternity	Extra‐pair paternity	Greater rates of extra‐pair paternity with increased breeding density, but not strictly with habitat type.	Not tested	Suburban vs. park (Breeding density)	Ryder et al. (2012)
	*Erithacus rubecula* (European robin)	Acoustic signal	Song characteristics	Males in noisy areas sang shorter songs with increased minimum frequency, lower complexity, and lower frequency bandwidth.	Plastic	Noise pollution	Montague et al. (2013)
	*Ficedula hypoleuca* (Pied flycatcher)	Extra‐pair paternity	Extra‐pair paternity	Greater polygyny and extra‐pair paternity in urban areas than in forests.	Not tested	Urban vs. rural	Grinkov et al. (2018)
	*Haemorhous mexicanus* (House finch)	Visual signal Behavior	Coloration Mate choice	Male coloration decreased along an urban gradient (yellower plumage in urban areas, redder plumage in rural areas). Females prefer local male coloration.	Not tested Not tested	Urban vs. rural	Giraudeau et al. (2018)
		Visual signal	Coloration	Urban birds are less colorful (more yellow, less red) and use carotenoids for coloration more efficiently than rural birds. After molting in the laboratory, urban and rural birds show no difference in coloration.	Plastic	Urban vs. rural (Resource availability)	Giraudeau et al. (2015)
		Visual signal Behavior	Coloration Aggression	Urban birds are less colorful than rural birds. Colorful, urban birds are less aggressive than drab urban and rural birds regardless of coloration.	Not tested Not tested	Urban vs. rural	Hasegawa et al. (2014)
		Acoustic signal	Song characteristics	Changes in bill shape selected by different resource use in urban vs. rural areas affect courtship song. Urban birds sang songs with slower trill rate, wider frequency range, and fewer note types.	Likely genetic	Urban vs. rural (Resource availability)	Badyaev et al. (2008)
	*Junco hyemalis* (Dark‐eyed junco)	Acoustic signal	Song characteristics Reproductive success	Urban males sang shorter songs with more atypical syllables than rural males. Within the urban site, males with more irregularities in their song had lower reproductive success.	Not tested	Urban vs. rural	Ferriera et al. (2016)
		Extra‐pair paternity Behavior Visual signal	Extra‐pair paternity Territorial aggression Coloration	Less extra‐pair paternity and more parental care in the urban population. Less territorial aggression in urban population, possibly due to differences in testosterone. Urban males are less colorful (less head black and tail white) than rural males.	Not tested Not tested Genetic	Urban vs. rural (Climate and resource availability)	Atwell et al. (2014)
	*Melospiza melodia* (Song sparrow)	Visual signal	Coloration Territorial aggression	Urban males have greater coloration (more chest melanin spotting) than rural males. Urban males that were more colorful were more aggressive, while more colorful rural males were less aggressive.	Not tested Not tested	Urban vs. rural	Beck et al. (2018)
	*Mimus polyglottos* (Northern mockingbird)	Acoustic signal Behavior	Vocal repertoire Territorial aggression	No difference in vocal repertoire between high and low lead‐contaminated areas. Males from high lead sites were more aggressive than birds captured from low lead areas.	NA Not tested	Metal pollution (lead)	McClelland et al. (2019)
	*Parus major* (Great tit)	Visual signal	Coloration	Birds living closer to roads and a source of metal pollution had reduced carotenoid coloration. There was no association between pollution and proximity to roads for melanin coloration.	Not tested	Metal pollution and roadways	Grunst et al. (2020)
		Visual signal	Coloration	Rural birds had larger “tie” melanin color patches than urban males.	Genetic	Urban vs. rural	Senar et al. (2014)
		Acoustic signal	Song characteristics	Males in noisy areas song songs with higher minimum frequency, longer duration, and more phrases.	Not tested	Noise pollution	Hamao et al. (2011)
		Acoustic signal	Mate choice	Females prefer lower frequency songs, but are more responsive to higher frequency songs in the presence of noise.	Plastic	Noise pollution	Halfwerk et al. (2011)
		Visual signal	Coloration	Rural birds had a greater hue (more yellow) in their plumage than urban birds.	Not tested	Urban vs. rural	Horak et al. (2004)
		Visual signal	Coloration	Rural birds had a greater hue (more yellow) in their plumage than urban birds.	Not tested	Urban vs. rural	Horak et al. (2001)
		Visual signal	Coloration	Urban‐born nestlings raised in rural habitats showed similar coloration to urban‐raised siblings. Rural‐born nestlings raised in urban habitats were less yellow than rural‐raised siblings.	Likely plastic	Urban vs. rural	Horak et al. (2000)
	Passerines	Visual signal	Sexually dimorphic coloration	Passerine species that had greater sexual dimorphism in coloration were less likely to colonize urban areas.	Not tested	Urban vs. rural	Iglesias‐Carrosco et al. (2019)
	*Pipilo maculatus* (Spotted towhee)	Extra‐pair paternity	Extra‐pair paternity	Probability of extra‐pair offspring in a nest was highest both near the edge of urban habitat and in interior of urban park.	Not tested	Habitat fragmentation	Bartos Smith et al. (2016)
	*Serinus canaria* (Canary)	Behavior	Mate choice	In noisy conditions, females showed reduced preferences for attractive male songs and laid fewer eggs.	Plastic	Noise pollution	des Aunay et al. (2017)
		Behavior	Mate choice	In noisy conditions, females do not express typical preferences (via copulation solicitation display) for low‐frequency songs.	Plastic	Noise pollution	des Aunay et al. (2014)
	*Taeniopygia guttata* (Zebra finch)	Acoustic signal	Song learning	Birds exposed to traffic noise during development learned songs with incorrect syntax, and possessed smaller brain regions associated with song learning than birds not exposed to traffic noise.	Plastic	Noise pollution	Potvin et al. (2016)
		Behavior	Mate choice Courtship	Strength of female preference for pair‐their bonded male decreased in noisy conditions. No difference in male courtship effort in relation to noise level.	Plastic Plastic	Noise pollution	Swaddle and Page (2007)
	*Troglodytes aedon musculus* (Southern house wren)	Acoustic signal	Song characteristics	Song amplitude was higher in noisier areas.	Not tested	Noise pollution	Sementili‐Cardoso and Donatelli (2021)
	*Turdus merula* (Eurasian blackbird)	Visual signal	Coloration	Higher frequency of leucism (white patches on melanic feathers) in urban areas.	Genetic	Urban vs. rural	Izquierdo et al. (2018)
	*Zonotrichia leucophyrs* (White‐crowned sparrow)	Acoustic signal	Song performance	Males had lower vocal performance in noisier areas.	Not tested	Noise pollution	Phillips et al. (2021)
		Acoustic signal	Song characteristics	Songs attenuated faster, particularly at higher frequencies, and reverberated more in urban areas. Urban songs had a faster trill rate, and shorter whistles than songs produced by rural males. There was no difference in dominant frequency or bandwidth between urban and rural songs.	Not tested	Urban vs. rural (impervious surfaces)	Phillips et al. (2020)
		Acoustic signal	Song characteristics	Syllable complexity, vocal performance, and minimum frequency of urban bird songs increased over ~50 years.	Not tested	Urban vs. rural (time lapse, noise pollution)	Moseley et al. (2019)
		Acoustic signal	Song learning	Males tutored in noisy conditions were more likely to learn a less‐masked song, sang at a higher frequency, and showed reduced vocal performance.	Plastic	Noise pollution	Moseley et al. (2018)
	*Zonotrichia leucophyrs nuttalli* (Nuttal's white‐crowned sparrow)	Behavior	Territorial aggression	Males approach playback speaker more closely in presence of noise, regardless of song characteristics. In general, males also approached playback speaker more closely when the songs had wider bandwidth.	Plastic	Noise pollution	Phillips and Derryberry (2018)
		Acoustic signal	Song characteristics	Males sing louder when experiencing noisier conditions. There was no difference in minimum frequency in association with noise levels.	Not tested	Noise pollution	Luther et al. (2017)
		Acoustic signal Behavior	Song characteristics Territorial defense	Males in noisier areas sing songs with higher minimum frequency, lower bandwidth, and decreased vocal performance. Typical bandwidth songs elicited the strongest responses; urban and rural males did not differ in this behavior. Song minimum frequency had no effect on male territorial response.	Not tested Plastic	Urban vs. rural (Noise pollution)	Luther et al. (2016)
Fish	*Cyprinella venusta* (Blacktail shiner)	Acoustic signal	Vocalizations	Traffic noise masks low‐frequency “growls” of fish.	NA	Noise pollution	Holt and Johnston (2015)
Mammal	*Pteropus policocephalus* (Grey‐headed flying fox)	Acoustic signal	Vocalizations	No difference between populations in vocalization characteristics.	NA	Urban vs. rural (noise)	Pearson and Clarke (2019)
Reptile	*Intellagama lesueurii* (Eastern water dragon)	Sexual size dimorphism	Sexual size dimorphism	More extreme sexual size dimorphism in rural habitats with greater conspecific density, compared to city parks.	Not tested	Urban vs. rural	Littleford‐Colquhoun et al. (2019)
	*Psammophilus dorsalis* (Indian rock agama)	Visual signal	Coloration	Males in urban (suburban) areas expressed duller coloration, and changed color more slowly during courtship interactions than rural males.	Not tested	Suburban vs. rural	Batabyal and Thaker (2017)
*Invertebrates*							
Arachnid	*Latrodectus hesperus* (Western black widow spider)	Behavior	Courtship Web building	No difference in latency to courtship behaviors among males reared at different temperatures. Females expressed greater web‐building behaviors at higher rearing temperature.	NA Plastic	Urban heat island	Johnson et al. (2020)
Insect	*Aquatica ficta* (Firefly)	Visual signal	Bioluminescence	When presented with low wavelength light, fireflies expressed brighter flash intensity and decreased flash frequency. There were no changes in response to long‐wavelength light, which the fireflies cannot perceive.	Plastic	Light pollution	Owens et al. (2018)
	*Chorthippus biguttulus* (Grasshopper)	Acoustic signal	Song characteristics	Males that experienced traffic noise during development sang higher frequency songs than those reared in quiet conditions. Males captured from the roadside sang higher frequency songs with and greater syllable to pause ratio than those captured from other areas.	Plasticity Possibly genetic	Noise pollution	Lampe et al. (2014)
		Acoustic signal	Song characteristics	Males captured near roadsides sang higher frequency songs with higher local frequency maxima.	Not tested	Noise pollution	Lampe et al. (2012)
	*Coenagrion puella* (Damselfly)	Locomotor performance	Mating success	Urban males had greater flight endurance; sexual selection in urban (but not rural) areas favors greater flight performance during scramble competition.	Not tested	Urban vs. rural (Habitat fragmentation)	Tüzün et al. (2017)
	*Drosophila melanogaster* (Fruit fly)	Behavior	Courtship	Fruit flies court for a longer duration of time before copulation when reared in light for three generations.	Not tested	Light pollution	McLay et al. (2018)
	*Gryllus bimaculatus* (Field cricket)	Behavior	Mate choice	Female crickets were less responsive to male calls in presence of noise, though not due to masking because of no spectral overlap between song and noise.	Plastic	Noise pollution	Schmidt et al. (2014)
	*Oecanthus pellucens* (Tree cricket)	Acoustic signal	Song characteristics	Males sang shorter calls and paused more in presence of traffic noise, but did not adjust song frequency or signal interval.	Plastic	Noise pollution	Orci et al. (2016)
	*Teleogryllus commodus* (Australian black field cricket)	Acoustic signal Behavior	Song characteristics Mate choice	No difference in male courtship songs between light rearing treatments. Females reared under high light were more likely to mate than those reared under control conditions.	NA Plastic	Light pollution	Botha et al. (2017)

Note

This table does not comprehensively cover all studies discussed in this paper but provides an overview of existing work at the interface of urbanization and sexual communication. We describe the traits investigated, focal aspect of urbanization and whether any observed shifts reflect plasticity or genetic changes.

Below, we highlight key themes in these studies and others that focused on environmental factors associated with urbanization (i.e., light, noise, and metal pollution, impervious surfaces, and urban heat islands) to synthesize our current understanding of how urbanization affects sexual communication and to identify important areas for future research.

## 4 ENVIRONMENTAL CONSEQUENCES OF URBANIZATION FOR SEXUAL COMMUNICATION AND SELECTION

**FIGURE 2:**
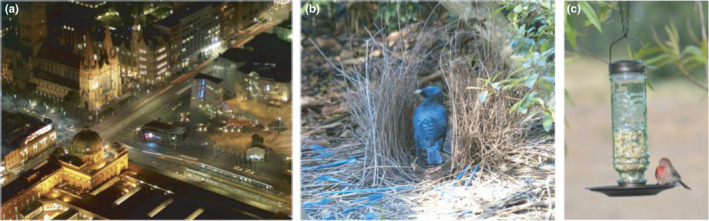
(a) Urban areas contain a suite of different environmental conditions relative to rural areas that are undisturbed by humans. Urban areas are polluted by artificial light, noise from traffic, different compositions of plants and animals, and contain large areas of impervious surface. Photo credit: Anne Aulsebrook. (b) Satin bowerbirds preferentially incorporate blue plastic waste, such as straws shown in the photograph, into their displays. (c) House finch at a birdfeeder. Urban and rural birds feed on different types of seeds, which have been selected for different beak shapes, ultimately causing divergence in song characteristics

Urbanization encompasses a complex suite of environmental changes, including pollution from noise, light, and metals; new physical structures that fragment habitats and change ambient conditions; and shifts in community structure and resource quality (Figure 2a). Below, we describe how sexual selection and sexual signaling can be affected by each of these environmental changes.

### 4.1 Ambient noise pollution

Cities experience much more background noise than rural areas, largely due to air and vehicle traffic (Barber et al., 2010). Traffic noise produces a low‐frequency sound that at least partially overlaps with the acoustic signals that many birds, frogs, and insects use to locate and attract potential mates (Brumm & Slabbekoorn, 2005). This frequency overlap is called auditory masking, or signal masking more generally, because it can obstruct the ability of individuals to detect and discriminate acoustic signals from background noise, thereby disrupting communication (Holt & Johnston, 2015). The effect of noise pollution on sexual communication has received a great deal of attention (including 58% of papers in Table 1), and much of that work has focused on birds (e.g., Patricelli & Blickley, 2006; Raboin & Elias, 2019; Slabbekoorn et al., 2018).

One of the most common findings is that birds increase the frequency of their songs in noisy urban areas (e.g., Brumm & Zollinger, 2013; Hamao et al., 2011; Lampe et al., 2012; Lampe et al., 2014; Luther et al., 2016; Montague et al., 2013; Moseley et al., 2018; Moseley et al., 2019; Parris et al., 2009; though see Rios‐Chelen et al., 2015). This has also been demonstrated in some other acoustically communicating taxa like frogs and grasshoppers (Lampe et al., 2012; Lampe et al., 2014; Schwartz & Bee, 2013; Tyack & Janik, 2013; but see Zollinger et al., 2012), though not as consistently. This discrepancy between taxa likely reflects physiological differences in both call production and detection. In general, increases in song frequency are considered adaptive because it avoids masking by a low‐frequency traffic noise. Such frequency shifts should increase detectability by receivers and make the acoustic signal more salient (Halfwerk et al., 2011; Wong & Lowry, 2016). However, it may come at the cost of receiver preferences and the use of the information encoded in songs. Lower frequency songs tend to be preferred by females and likely serve as honest indicators of male condition, so a consistent shift to higher frequency songs may disrupt sexual selection (e.g., Halfwerk et al., 2011).

Indeed, females experiencing anthropogenic noise often show weaker preferences for male traits (des Aunay et al., 2014, 2017; Bent et al., 2018; Swaddle & Page, 2007). For example, the monogamous zebra finch (*Taeniopygia guttata*) showed a weaker preference for pair‐bonded males when exposed to high‐amplitude white noise, suggesting that normally monogamous songbird populations may show more extra‐pair behavior in areas with more anthropogenic noise (Swaddle & Page, 2007). In canaries (*Serinus canaria*), females do not express typical preferences for low‐frequency male songs in noisy conditions, thereby weakening sexual selection (des Aunay et al., 2014, 2017). If preferred low‐frequency signals are consistently masked, it is unclear whether females will begin preferring higher frequency songs (thereby disrupting signal‐cost associations and underlying signal honesty), whether sexual selection will weaken in the population because males rarely produce preferred signals (des Aunay et al., 2014), or whether females will shift the relative importance of other signal modalities during mate choice (Partan, 2013; Rios‐Chelen et al., 2015). Future research should explore whether the capacity for flexible acoustic communication influences the potential for taxa to persist in noisy urban areas.

It is important to note that not all species adjust signals and responses in the same way, if at all, to low‐frequency noise pollution. A recent meta‐analysis discovered that birds seem better than frogs at adjusting song frequencies to avoid signal masking (Roca et al., 2016). This may be due to physiological constraints associated with how sound is produced in these taxa, or perhaps to other behavioral differences that improve signal detection. For example, frogs may be more likely to increase call amplitude, rather than frequency, to enhance signal transmission (Roca et al., 2016). Consistent with this idea, *Oecanthus pellucens* tree crickets did not adjust the frequency of their songs, but instead performed shorter calls with more pauses in the presence of vehicle traffic (Orci et al., 2016). In addition, not all noise arising from urbanization leads to interference with the perception of acoustic signals. Many acoustically signaling organisms have finely tuned hearing and sound production apparatuses that are unable to detect sounds in different frequencies (Dusenbery, 1992). For example, *Eleuthereodactylus coqui* is an invasive species of frog on the Big Island of Hawaii whose extremely loud call has dramatically altered the island's soundscape (Woolbright et al., 2006). However, there is no evidence that their loud calls interfere with an acoustically communicating cricket species, *Teleogryllus oceanicus*, that occurs in similar habitats on the island. There is no interference because the frequency of the frog call does not overlap with the crickets' peak range of auditory perception (Zuk et al., 2017). Similarly, female *Gryllus bimaculatus* crickets responded less to male songs played alongside noise, but this is not due to masking because the experimental noise fell outside the range that crickets are able to perceive (Schmidt et al., 2014). Such variation in physiology and sensory ecology makes it difficult to generalize about the effects of low‐frequency traffic or other noise across taxa, since some forms of anthropogenic noise may have little effect on some species and completely interfere with signaling in others.

Noise pollution can also alter traits not involved in acoustic communication by inducing a stress response (Ising & Kruppa, 2004), making individuals more susceptible to disease and parasitism (Masud et al., 2020). For example, Troianowski et al. (2017) found that experimental noise exposure increased stress hormone levels and induced immunosuppression in *Hyla arborea* (European tree frog), which in turn reduced the intensity of coloration involved in sexual signaling. This is important because it shows that noisy urban conditions can disrupt non‐acoustic animal communication. Birds reared under noisy conditions have also been shown to reduce investment in brain regions associated with song learning (Ferriera et al., 2016; Potvin et al., 2016). In this case, songs did not change directly in response to noise pollution, but rather due to cascading physiological changes caused by noise exposure.

Another important issue with research on the masking effects of anthropogenic noise is that studies often do not include non‐anthropogenic noise treatments as controls (e.g., conspecific choruses, biological noise from other species, or other natural background sounds like waterfalls or ocean waves; Bee & Swanson, 2007; Davidson et al., 2017). Studies designed to isolate specific responses to anthropogenic noise (i.e., including natural sound controls) are critical for understanding and potentially mediating the aspects of urban environments that disrupt acoustic sexual signaling (Gough et al., 2014). A few studies can serve as models for how to address this issue. Bee and Swanson (2007) examined whether female *Hyla chrysoscelis* treefrogs in Minnesota differentially responded to male calls in the presence of traffic noise, a conspecific chorus, or no noise. They found that females responded similarly to the anthropogenic and biological noise by responding to calls more slowly. *Odontophrynus americanus* frogs in Argentina adjust their calls in response to both traffic and chorus noise, but the particular shifts in call parameters depended on the type of noise experienced (Grenat et al., 2019). Understanding the nature and specificity of responses to different anthropogenic noises will help researchers predict the potential additive and interactive effects of multiple noise sources.

### 4.2 Artificial light pollution

Artificial light at night, or ALAN, from streetlamps and other sources of anthropogenic illumination, has dramatically increased with the expansion of urban areas (Bennie et al., 2015), and recent studies suggest that it poses one of the greatest threats to biodiversity (Davies & Smyth, 2018; Hölker et al., 2010; Lewis et al., 2020). Illumination plays an important role in regulating physiology and behavior of organisms, but the impact of ALAN has only recently begun to receive significant research attention (Longcore & Rich, 2004; Swaddle et al., 2015). To date, research on biological impacts of ALAN has focused on physiological and ecological, rather than evolutionary, considerations (e.g., Desouhant et al., 2019; Jones et al., 2015; McLay et al., 2017, 2018). However, ALAN has the potential to foster widespread and rapid evolutionary change both because of its intensity and geographic extent and because it creates truly novel environmental conditions for many taxa (Hopkins et al., 2018; Swaddle et al., 2015). Regarding mating outcomes, ALAN has the potential to both disrupt visual signaling, and affect other behavioral and physiological aspects of reproduction (Longcore & Rich, 2004).

ALAN can directly obstruct sexual communication for nocturnal animals that use visual signaling. For example, it interferes with bioluminescent mating signals by fireflies (Firebaugh & Haynes, 2016, 2019). Two firefly species, *Photuris versicolor* and *Photinus pyralis*, were both lured to artificial LED light, and were less likely to signal in artificially illuminated areas than unlit outdoor spaces (Firebaugh & Haynes, 2019). *Photinus pyralis* also experienced reduced mating success in lit areas relative to dark habitats, suggesting that light‐polluted areas may act as demographic traps (Firebaugh & Haynes, 2019; Owens & Lewis, 2018). Similarly, female glow‐worms (*Lampyris noctiluca*) do not move away from artificial light even though it significantly reduces mate attraction, suggesting a maladaptive response (Elgert et al., 2020). Most studies so far suggest that ALAN exerts negative fitness consequences on nocturnal insects (Mbugua et al., 2020; Owens & Lewis, 2018). These results and the rapid spread of ALAN across the landscape indicate that there is a pressing need for research to determine whether signal expression or detection in bioluminescent organisms like fireflies can evolve to avoid masking and allow for adaptation to artificial light. Behavioral responses to light stimuli may be less flexible than responses to acoustic signals, reducing the opportunity for adaptation.

Similar to noise pollution, ALAN may affect the strength of sexual selection in ways other than direct interference with signal detection, including creating stressful conditions that affect physiology and behavior. ALAN disrupts normal day‐night cues, which can cause other cascading shifts in animal physiology, behavior, and reproductive success (Desouhant et al., 2019; Jones et al., 2015). Such negative non‐communication impacts are illustrated by McLay et al. (2017), who found that *Drosophila melanogaster* reared under high light conditions for several generations show reduced fecundity. Females exposed to even the lowest level of artificial light exhibited reduced oviposition (McLay et al., 2017), highlighting the significant effects that ALAN can have on reproductive success and population viability through disruption of circadian rhythms. Some female crickets reared with chronic exposure to ALAN are less discriminating during mating interactions and more likely to mate than females reared under typical day‐night light cues, even though there was no difference in male song characteristics in association with ALAN exposure (Botha et al., 2017). Less stringent female mating preferences will weaken sexual selection in a population, and negate benefits that females may gain (directly or indirectly) by being choosey (Zuk & Simmons, 2018). In the same study, male crickets reared under certain illumination conditions were also rejected more often by females. The authors speculated that the difference may be due to the differential expression of cuticular hydrocarbons, a chemical signal used by insects during mating decisions (Botha et al., 2017). Similarly, female *Physalaemus pustulosus* (Tungara frogs) are less selective about male signal quality (i.e., acoustic call characteristics) under illuminated conditions (Rand et al., 1997). The authors suspected that predation risk is greater under higher light levels, contributing to the behavioral shift. These studies suggest that ALAN may generally cause a weakening of sexual selection, even in organisms that do not use visual cues during sexual communication (though see Underhill & Höbel, 2018).

ALAN likely has diverse, taxon‐specific impacts on sexual communication, and thus should provide evolutionary biologists with opportunities to better understand how signal masking and disruption generally impact sexual selection. ALAN can affect the behavior and physiology of both nocturnal and diurnal organisms (Davies & Smyth, 2018), but the practical implications will depend on the extent to which organisms are exposed to ALAN. Understanding the evolutionary impact of ALAN signaling disruption can also guide urban conservation measures. For example, *Aquatica ficta* firefly males emit brighter signals with decreased frequency when exposed to artificial light at shorter wavelengths (<533 nm) but show no response to longer wavelength (≥597 nm) ambient light, suggesting that replacing broad‐spectrum lighting with longer wavelength lighting could help this species (and maybe other fireflies) thrive in urban settings (Owens et al., 2018).

### 4.3 Metal pollution

Heavy metals, particularly lead, tend to accumulate in urban areas from air pollution and from historical use in products like gasoline and house paint (Gulson et al., 1995). Such toxins can induce stress responses in many organisms (Candolin & Wong, 2019; Isaksson, 2015). Because many sexual signals are condition‐dependent, physiological stress tends to negatively affect sexual signal intensity (Hutton & McGraw, 2016). In general, lead exposure appears to reduce the signal intensity in wild individuals across pollution gradients, and also during experimental exposure in the laboratory. For example, great tits (*Parus major*) express reduced carotenoid‐based coloration when living near sources of heavy metal pollution (Grunst et al., 2020), and pigeons had less colorful plumage when exposed to lead (Chatelain et al., 2017). Population viability may decrease if males do not exhibit high‐quality signals that are preferred by females. So far, it is unclear whether female preferences for male color traits are also impacted by heavy metal exposure.

Effects of metal pollution on sexual signaling are not consistently negative and appear to depend on the particular compound. A *Bufo raddei* frog population in a polluted region of China exhibited higher reproductive investment (i.e., more complex songs, stronger amplexus force, larger breeding glands) relative to conspecifics in less‐polluted areas at the cost of survival (Guo et al., 2018). This may reflect a shift to investment in reproduction in response to stressful conditions. If frogs from polluted areas reproduce more than those from less impacted areas, it should accelerate the rate of adaptation because more individuals that can tolerate such conditions will comprise a greater portion of the population. In *Mimus polyglottos* mockingbirds, song repertoires in lead‐polluted urban populations did not differ from populations experiencing lower levels of lead contamination (McClelland et al., 2019). Other metals such as copper and zinc can also show positive, or at least mixed, effects on signal expression when experienced in small doses (Chatelain et al., 2017; Giraudeau et al., 2015). These strong but diverse responses suggest that more research is needed to identify mechanisms linking specific contaminants to aspects of sexual signaling across a range of taxa.

### 4.4 Fragmented habitats

Construction of roads and buildings in urban areas not only results in habitat loss, but also creates more edges and smaller habitat patches. Although fragmentation can have diverse impacts on resource availability (Zanette et al., 2000), smaller fragments have proportionally more edge habitats that support more nectar, seed, and fruits resources used by some birds (Green, 1984) and may also have more human‐created food sources (Robb et al., 2008), which can in turn affect condition‐dependent signaling. For example, for *Pipilo maculatus* (spotted towhee) in urban parks in Oregon (USA), females closest to the edges were in the best condition and had the longest tail length because these areas were closer to anthropogenic food sources like bird feeders (Bartos Smith et al., 2016). Resource distribution also appeared to affect the prevalence of multiple mating. Extra‐pair paternity was highest both at the habitat edge where food is most abundant and in the interior of habitat patches (it was lowest at intermediate distances from habitat edges). The authors suggest that females living in the interior of the habitat encounter more males when searching for food (Bartos Smith et al., 2016).

Habitat fragmentation can also alter mating systems by changing the distance required to find mates or suitable breeding habitat. In *Coenagrion puella* damselflies, urban habitat fragmentation has generated selection for greater flight performance (Tüzün et al., 2017). Males in cities need to fly farther to reach the breeding ponds, and greater flight endurance was also associated with greater mating success during scramble competition (Tüzün et al., 2017). In this case, urbanization is correlated with favorable traits that actually increase reproductive success. If variation in flight endurance traits is heritable, this trait should quickly evolve in the population and facilitate rapid adaptation to cities.

### 4.5 Impervious surfaces

Impervious surfaces in cities retain heat, contributing to what is known as the urban heat island effect (Santamouris, 2015). Increased temperatures in urban areas can exacerbate warming that occurs due to climate change, but this urban phenomenon is distinct because temperatures do not fall as much overnight as they do in rural areas lacking human structures. Increased temperatures caused by urban heat islands are particularly likely to affect volatile pheromone‐based sexual signaling, which can have consequences for mate recognition, mate choice, and population viability (Henneken & Jones, 2017). Thermal stress influences several aspects of chemical signaling, including pheromone synthesis, persistence after emission, and detectability (Groot & Zizzari, 2019). It can also affect the perceived quality of the individual producing the chemical signal. For example, in the beewolf (*Philanthus triangulum*), higher temperatures during development are associated with greater pheromone production typical of higher quality males (Roeser‐Mueller et al., 2010). Although it is possible that higher temperatures during development also increase beewolf condition, Roeser‐Mueller et al. (2010) suggest that higher temperatures actually disrupt honest signaling relationships that exist in cooler temperatures for these insects because even poor‐quality males can produce high‐quality signals.

The mating systems of insects and other ectothermic animals may be particularly affected by urban heat islands because their behavior and physiology are highly sensitive to temperature (Suzaki et al., 2018). For example, female Western black widow spiders (*Latrodectus hesperus*) engage in greater web‐building behaviors at higher temperatures (Johnson et al., 2020). Svensson et al. (2020) show that temperature has dramatic effects on the strength of sexual conflict and maintenance of a reproductive polymorphism in *Ischnura* spp. damselflies. A male‐mimic female morph that circumvents male harassment is sustained at higher frequencies in populations experiencing cooler temperatures, likely because they develop faster and are less sensitive to temperature than other morphs (Svensson et al., 2020; Takahashi et al., 2014). The locus controlling expression of the female morph is also highly pleiotropic, affecting various physiological and reproductive traits and appears to interact with other loci that affect thermal adaptation (Svensson et al., 2020; Willink et al., 2020). The Svensson et al. (2020) study not only emphasizes that temperature can strongly affect mating system dynamics, but also that organisms may exhibit interactions between environmental conditions and genetics that shape their potential for adaptation to novel urban conditions.

Impervious surfaces and other urban structures have other, underappreciated effects that may also affect sexual signaling. The physical structures of cities can interfere with long‐distance acoustic and visual signaling by obstructing the signal pathway, reducing the distance that the signal can travel and be received (Phillips et al., 2020; Slabbekoorn et al., 2018). For example, white‐crowned sparrow (*Zonotrichia leucophyrs*) songs attenuated faster and reverberated more in urban areas (Phillips et al., 2020). However, some species may actually take advantage of human‐made structures to amplify signals. *Anurogryllus muticus*, short‐tailed crickets in Panama, produced louder, more intense songs when singing near human structures than when calling in more natural habitats like grass or leaf litter because building walls amplify calls (Erregger & Schmidt, 2018). Similarly, male Mientien tree frogs, *Kurixalus idiootocus*, prefer calling inside rather than outside of storm drains, and calls from within storm drains have higher amplitude and note duration compared to outside calls (Tan et al., 2014). This use of habitat to amplify sound is similar to behaviors in other taxa in natural habitats (e.g., *Metaphrynella sundana* tree‐hole frogs exploit resonance effects of tree holes; Lardner & Lakim, 2002). It is not entirely clear if this amplification is adaptive, though. Louder calls may attract more mates, but it may also make the caller more vulnerable to exploitation by eavesdropping predators. These examples also illustrate how research on responses to natural analogs of urban features (e.g., tree holes vs. storm drains) may provide insight into adaptation and community assembly in urban environments.

### 4.6 Human refuse

Human materials are used by some species to enhance visual displays. Bowerbirds (*Ptilonorhynchus nuchalis* and *P*. *violaceus*) use colorful plastic items like straws, bottle rings, wires, and other human‐associated items in their displays (Doerr, 2010; Wojcieszek et al., 2006). For example, blue plastic bottle tops were the most popular environmental adornment, relative to their availability, on bowers of male *P*. *violaceus* (Figure 1b; Wojcieszek et al., 2006, 2007). It is possible that blue items exploit a pre‐existing bias in females, or that blue items are rare in pristine forests and thus their accumulation may honestly advertise male quality (Umbers, 2013). It is unclear whether the introduction of blue, and other colored, anthropogenic litter disrupts honest signaling in bowerbirds and negatively impacts population fitness.

Human refuse can also disrupt sexual communication. One classic example is *Julodimorpha bakeweßlli* beetles that attempt to mate with beer bottles (Gwynne & Rentz, 1983). In this study, the authors speculated that the reflective sheen of bottles provides a supernormal stimulus for males because it resembles the elytra color and markings of large female beetles. Discarded bottles might significantly disrupt the mating system in this species and reduce population fitness due to lack of successful reproduction.

Chemical refuse in cities may broadly disrupt sexual communication. Human pest control often uses synthetic materials to exploit chemical or visual mating cues of insects (e.g., Chen et al., 2014), suggesting the potential for human products to disrupt sexual communication. Endocrine‐disrupting chemicals (EDCs), mostly released from the manufacture and use of anthropogenic materials, are commonly found in urban sewage effluent (Kumar et al., 2020) and are known to have widespread impacts on male mating behavior (Bertram et al., 2020). The potential impacts of EDCs on sexual communication in urban animals should provide many opportunities for important research in the coming years.

### 4.7 Changes in resource quality and availability

Cities often possess dramatically different patterns of biodiversity, productivity, and nutrient availability relative to areas less impacted by humans (Grimm et al., 2008; Shochat et al., 2006). For example, vegetation in urban areas often includes exotic and non‐native plant species used for landscaping and gardening. For many animals, cities offer greater food and nutrient abundance due to human activities like bird feeder supplementation, fertilizer use, and discarded food scraps. This anthropogenic influx of resources can reduce variation in the quality of territories or nesting areas, as well as the expression of condition‐dependent signals (Chace & Walsh, 2006; Tryjanowski et al., 2015). Such altered resource availability in cities can create an unreliable relationship between a sexual signal and its information content because individuals can produce a high‐quality signal regardless of their underlying quality (Higgins & Reader, 2009; Snell‐Rood et al., 2015). If most potential mates express high‐quality sexual signals, sexual selection will weaken in a population because most males meet female preference standards.

A series of studies on Northern cardinals (*Cardinalis cardinalis*) provides a compelling case study of how urban‐associated resource shifts can create an evolutionary trap (Schlaepfer et al., 2002), where formerly adaptive behaviors become maladaptive under new conditions (Borgmann & Rodewald, 2004; Narango & Rodewald, 2018; Rodewald et al., 2011). Male coloration is used as a sexual signal, and the intensity of expressed coloration depends on access to carotenoid‐rich foods during the molt. An exotic honeysuckle shrub (*Lonicera* spp.) present in rural areas produces carotenoid‐rich fruit that is highly abundant during the molting season. Though this plant allows males to express high levels of coloration, cardinals that nest in this shrub experience much higher levels of predation (Borgmann & Rodewald, 2004). Though brightly colored males typically experience high fitness, they experience lower reproductive success when nesting the invasive honeysuckle plants due to increased predation. Interestingly, this evolutionary trap does not occur in urban areas. This is not because urban populations are better adapted, but instead because they experience a breakdown of the associations among coloration, territory quality, breeding phenology, and reproductive success (Rodewald et al., 2011).

Differences in food resources between urban and rural populations might be particularly likely to favor divergence in such traits that contribute to reproductive isolation. Evolutionary differences in traits that are important for both survival and reproduction, or “magic” traits, are effective at promoting reproductive isolation between ecologically dissimilar environments because they simultaneously facilitate local adaptation and reproductive isolation (Schluter, 2000; Servedio et al., 2011). One of the best examples of such synergistic divergent selection comes from house finches (*Haemorhous mexicanus*). In Arizona, these birds feed on small seeds in the desert, but have access to large, hard sunflower seeds from bird feeders in urban areas (Figure 2c). Divergent selection on bite force has driven evolutionary differences in beak size and shape between urban and rural environments (Badyaev et al., 2008). As a byproduct of the divergence in beak morphology, courtship song has also shifted between urban and rural house finches. Urban birds with longer, deeper beaks sing songs with a wider frequency range, fewer types of notes, and slower trills compared to rural birds that have shorter, shallower beaks (Badyaev et al., 2008). There is evidence of genetic differentiation among urban and rural populations in this system despite close geographic proximity, suggesting assortative mating based on habitat of origin. Over time, the buildup of different sexual signals and preferences can further contribute to reproductive isolation between urban and rural environments (Halfwerk, 2021).

Pollution and other urban stressors may interact with food resources and other biotic conditions to affect sexual signaling. Such interactive effects are illustrated by studies on house finches, a model system for urban sexual communication research. Male coloration is considered an honest indicator of individual quality (e.g., health, condition) under natural conditions because carotenoids cannot be synthesized de novo and instead must be obtained through the diet (Hill, 1991; Hill & Montgomerie, 1994). House finches in cities are less colorful and have yellower plumage, while those in rural areas are more colorful and have redder feathers (Giraudeau et al., 2015, 2018; Hasegawa et al., 2014). Neither experimentally manipulated carotenoid access nor exposure to oxidative stress could explain differences in plumage coloration between urban and desert house finches (Giraudeau et al., 2015). Environmental differences are most likely driving the differences in coloration between urban and rural birds (Giraudeau et al., 2015), and may be associated with the prevalence of certain microorganisms (Giraudeau et al., 2016) rather than carotenoid content of food. Female preferences appear to track male color differences between populations, providing a rare example of flexible female preferences that reflect urban‐associated changes in male signal expression (Giraudeau et al., 2018). Simultaneous divergence of male signals and female preferences can facilitate population‐level divergence in phenotypes, and can represent the first steps of speciation (Servedio & Boughman, 2017). These results illustrate the potential for urban studies to serve as natural experiments that further the general understanding of sexual communication diversification and the possible establishment of reproductive barriers.

### 4.8 Shift in predation pressure

To our knowledge, there appear to be no studies that have explicitly investigated how changes in predation regimes impact sexual communication in urban areas. However, these impacts are likely common. Urbanization alters predator–prey interactions for many species by reducing the prevalence of specialist species and those at higher trophic levels (Burkman & Gardiner, 2014; Raupp et al., 2010). Whether predation risk becomes more or less intense in urban areas will depend on the particular species and other characteristics of the ecosystem. For example, the desert ecosystem outside of Phoenix, AZ (USA) is typically characterized by limited resources. Resources are more abundant in the city which supports greater arthropod communities; this causes predation of arthropods by birds to become the dominant force in trophic dynamics in this urban area (Faeth et al., 2005). In general, not just in the desert, birds in urban areas appear to face reduced predation pressure due to a lack of top predators, while arthropods likely experience stronger predation due to increased bird densities (Gering & Blair, 1999; Shochat et al., 2006).

Predation risk has well‐documented effects on the evolution of sexual communication in natural settings by affecting the conspicuousness of signals, strength of female preferences, and general sexual selection dynamics (Martin et al., 2014; Sarno et al., 2017; Stanger‐Hall & Lloyd, 2015; Tuttle & Ryan, 1981; Zuk & Kolluru, 1998). Therefore, urban environments should provide excellent opportunities for exploring the influence of predation on sexual signaling in a range of taxa because of the relatively recent change in selection pressures, and different levels of predation risk in urban and rural environments. Because a large body of work has focused on how sexual selection responds to predation risk in the wild, we can use this existing knowledge to generate predictions for how sexual communication changes in urban environments. Such work can shed light on how quickly and predictably traits involved in sexual communication can respond to changes in selection pressures.

## 5 ADAPTIVE VERSUS MALADAPTIVE TRAIT CHANGE

**FIGURE 3:**
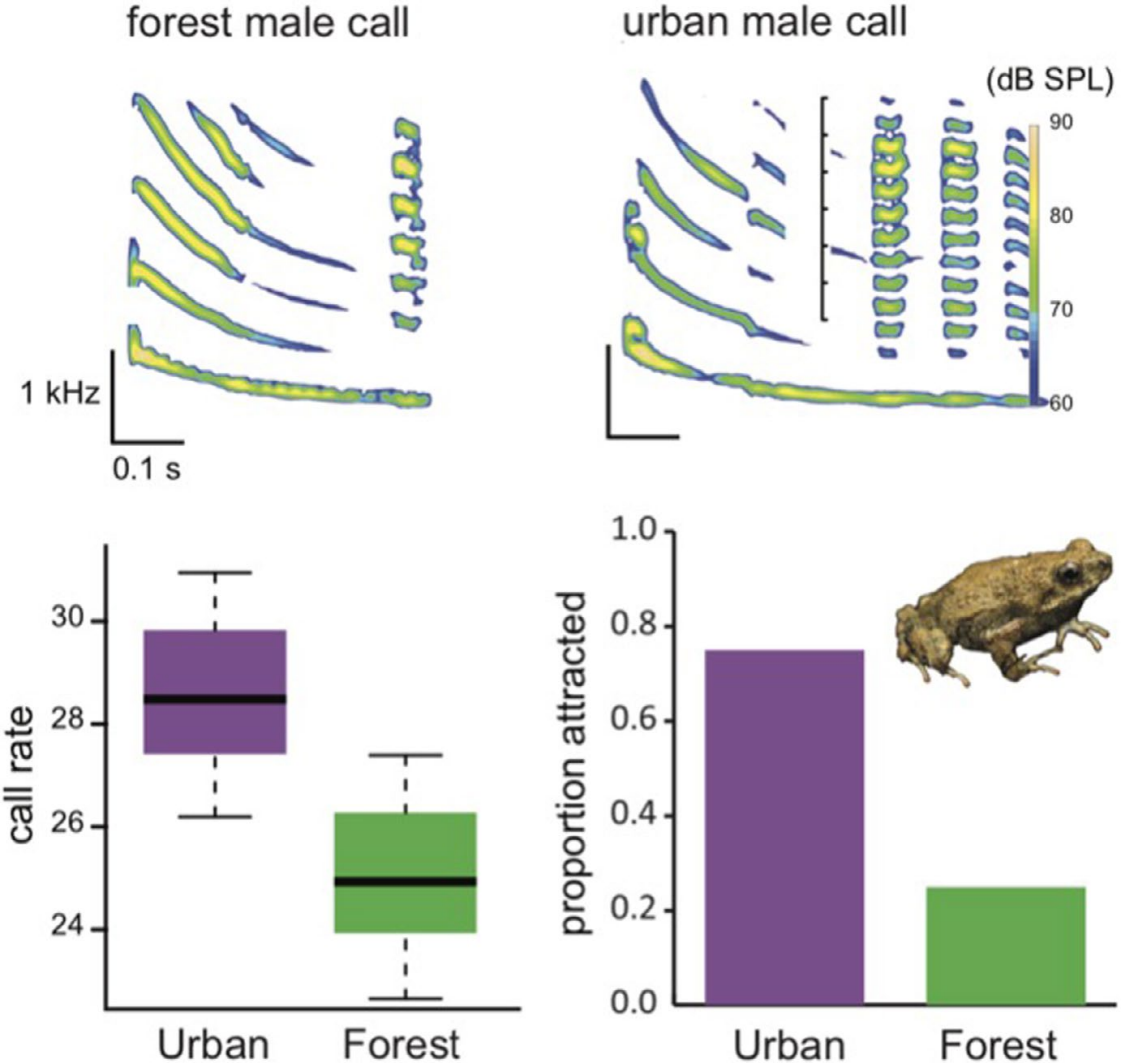
Male Tungara frogs performed faster, more elaborate calls than counterparts from rural areas. These more complex urban calls are adaptive and attracted more females than the rural call. Adapted from Halfwerk et al. (2019)

Overall, we found relatively few unambiguous cases of sexual communication traits adaptively responding to urban environments. Perhaps the best‐documented example is Tungara frogs (*Physalaemus pustulosus*) in Panama (Halfwerk et al., 2019). In urban areas—where there is pollution from noise and light, lower risk of predation, and stronger competition for mates—male calls are faster and more elaborate than those in rural areas (Figure 3; Halfwerk et al., 2019). Females from both urban and rural populations preferred the calls of urban males, showing that this change is advantageous during sexual selection. Because frog predation risk is much lower in urban areas, this more conspicuous call does not generate the same costs as in forested areas. This adaptive shift in sexual signaling reflects phenotypic flexibility—urban males, when translocated to the forest, shift their calls in accordance to local predation conditions and produce songs more like rural males (Halfwerk et al., 2019). This is likely the first study to characterize trait divergence between rural and urban populations, and also assess how sexual and natural selection respond to urban‐associated traits.

The field needs more studies that take a comprehensive approach to understand whether differences in trait expression between urban and rural populations positively or negatively affect reproductive fitness. Many studies document a “not sexy in the city” effect where signal expression is reduced (e.g., Batabyal & Thaker, 2017; Hasegawa et al., 2014; Horak et al., 2001, 2004; Potvin et al., 2016), or the relationship between signal expression and information content is disrupted (Tringali & Bowman, 2015). However, studies often characterize trait expression, but do not also investigate female preferences for modified signals (though see des Aunay et al., 2014; Botha et al., 2017; Giraudeau et al., 2018; Swaddle & Page, 2007) or how natural selection acts on these modified traits (e.g., through signal exploitation by natural enemies, Erregger & Schmidt, 2018; Zuk & Kolluru, 1998). For instance, despite an abundance of studies showing frequency shifts in bird songs in response to anthropogenic noise, in most cases it remains unclear whether such shifts actually improve signal transmission (Nemeth & Brumm, 2010), how females respond to these higher frequency songs, or whether changes in signal transmission affect reproductive success (Narango & Rodewald, 2018). Though singing at a higher frequency should ameliorate signal masking, it does not guarantee greater reproductive success. Understanding whether signaling changes are adaptive or not will be important for predicting whether populations are resilient and able to adapt to new conditions.

## 6 PLASTIC VERSUS EVOLUTIONARY CHANGES IN RESPONSE TO URBAN ENVIRONMENTS

Understanding the mechanism underlying responses to urbanization represents an important priority for research focused on sexual communication in urban settings. Though many studies document differences in sexually selected traits between urban and rural habitats, it is rarely tested whether the observed shifts reflect evolutionary changes or phenotypic plasticity (Table 1). The first logical step is to understand whether traits indeed differ between urban and rural environments, and the field has established a solid baseline in this regard. Particularly in regard to noise, light, and metal pollution, we are beginning to gain a fairly robust understanding of plastic responses to these urban stressors because they can be experimentally manipulated in laboratory or field studies (e.g., Chatelain et al., 2017; des Aunay et al., 2014; Halfwerk et al., 2011; Owens et al., 2018). In general, few studies have attempted to determine whether observed population differences in sexual trait expression between urban and rural environments are heritable. Urban evolution research is relatively young, and there are logistical challenges to understanding the mechanism underlying trait divergence. In addition, urbanization is a relatively new phenomenon in evolutionary history and these selection pressures are largely novel. In many cases, the organisms are difficult to breed in captivity under common garden conditions, or there simply has not been sufficient time to investigate evolutionary questions in wild animals with long generation times. However, it is critical to understand the extent to which novel selection pressures in urban environments lead to heritable changes, as this affects the potential for adaptation.

Plastic trait changes can have important ecological and evolutionary effects on populations (Miner et al., 2005), and phenotypic plasticity may often represent a precursor to, and possibly even facilitate, evolutionary change via genetic accommodation (Levis & Pfennig, 2016). In particular, behavioral flexibility may serve as an important early mechanism of adaptation to novel conditions and pave the way to evolutionary change (Gordon & Uetz, 2011; Snell‐Rood, 2013; Zuk et al., 2014). Compared to other traits, behavior is extremely responsive to the environment and able to change over very short timescales. As such, facultative shifts in sexual behavior can be a powerful way for animals to successfully cope with novel conditions associated with urbanization because they do not necessarily require genetic changes (Zuk et al., 2014). For instance, *Teleogryllus oceanicus* (Pacific field crickets) represents one of the few documented examples of the rapid evolution of a sexual signal (Svensson, 2019; Svensson & Gosden, 2007). These crickets have lost the ability to sing in response to a parasitoid fly due to a mutation that affects wing morphology (Zuk et al., 2006). Plasticity in male alternative mating behaviors and strength of female preferences appears to have been critical in allowing population persistence in the face of a novel environmental perturbation (Bailey & Zuk, 2008; Heinen‐Kay & Zuk, 2019; Tinghitella et al., 2009). Distinguishing flexible trait expression from genetic changes in a population can provide more insight into the potential for adaptive evolution in the face of urbanization and the extent to which such changes may lead to speciation events.

A few studies have discovered likely instances of evolution in response to urbanization. The most unambiguous case of sexual signal evolution is the dark‐eyed junco (*Junco hyemalis*). These birds have historically lived in rural mountain habitats, but recently found a population in the suburban region of San Diego (Yeh & Price, 2004). Males in the new, urban population exhibit reduced coloration relative to the rural population; this difference in coloration is genetically based and unlikely to be explained by drift or founder effects (Atwell et al., 2014; Yeh, 2004). However, for these junco populations, there are major differences in the climate, as well as urbanization, so it is not entirely clear which selective forces are responsible for divergence in male coloration. There are several suggestive cases of urban evolution of sexually selected traits. For example, in a reciprocal transplant study, grasshoppers (*Chorthippus biguttulus*) captured from roadsides as nymphs and reared under common conditions produced higher frequency songs compared to those captured from quieter habitats (Lampe et al., 2012, 2014). A few studies in birds have also identified trait differences that likely have a genetic basis. The beak shape, and consequently song characteristics, of house finches in Arizona, also represents a likely case of evolution in response to urban‐associated differences in food resources (Badyaev et al., 2008). These birds show distinct beak development quite early during embryonic development, and urban and rural populations are genetically distinct (Badyaev et al., 2008). There are also a few color traits in birds caused by a genetic mutation that differs in its representation in urban versus rural areas (Izquierdo et al., 2018; Senar et al., 2014). However, the role of these bird color traits during sexual selection is not well understood.

Sexual traits that are adaptive in urban environments should evolve quickly if they are associated with reproductive success—the trait should spread quickly in the population because individuals bearing the trait should experience greater reproductive success. Yet surprisingly, there are few documented examples of sexual evolution in a contemporary timeframe (Svensson, 2019; Svensson & Gosden, 2007; Zuk & Tinghitella, 2008). Many studies have shown differences in signal expression and mating behaviors between urban and rural environments, but it is often unclear, or untested, whether observed differences have a genetic basis or result from phenotypic plasticity. More research is needed to determine if the rapid evolution of sexual signals, as opposed to plastic shifts, is indeed rare or whether the topic has not yet been adequately investigated. Many of the studies we reviewed test for differences in signal expression and behavior between wild‐caught individuals experiencing different environmental conditions, and thus were not designed to assess whether differences exist because of evolution or plasticity. To make this distinction, we need more reciprocal transplant and common garden experiments aimed at understanding mechanisms connecting environmental changes to phenotypic differences between urban and non‐urban residents. Long‐term studies in rapidly developing areas would also provide excellent opportunities for better understanding adaptation to urbanization. The issue of reporting phenotypic differences but not testing whether they are due to evolution or plasticity is not unique to sexual traits, but is broadly a limitation in the new field of urban evolution (Alberti et al., 2017). It is logistically much easier and indeed represents an important first step, to determine whether differences in trait expression exist between populations in urban versus rural environments. Elucidating whether and under what circumstances signal responses to urbanization reflect rapid evolution will be critical for determining the potential for species to adapt to increasingly human‐dominated environments.

## 7 CONCLUDING REMARKS—THE FUTURE OF URBAN SEXUAL COMMUNICATION RESEARCH

### 7.1 Natural experiments to test and update evolutionary theory

Urbanization, considered the most irreversible form of human‐driven land‐use change, continues to expand rapidly around the globe. Researchers increasingly recognize the value of studying how urbanization impacts evolution, both because understanding urban evolution may help researchers predict and mitigate its effects and because urbanization provides a natural experiment that creates exciting opportunities for generalizing theory in the field (Thompson et al., 2018). Researchers should take advantage of these ongoing, replicated natural experiments to address basic questions about how the environment interacts with sexual communication (see below). The field of urban ecology has grown tremendously over the last several decades because ecologists have recognized the utility of natural experiments caused by human impacts (McDonnell & Pickett, 1990; Tanner et al., 2014), and recent calls suggest the field of evolutionary biology should also capitalize on these natural experiments (Santangelo et al., 2018). While growing attention has been paid to understanding evolution by natural selection in urban areas, there has so far been too little focus on sexual selection (though see Senar et al., 2014; Sepp et al., 2020).

Moreover, most research that has been done on urban sexual selection has focused on single components of the urban environment (e.g., noise pollution). However, much of the novelty of urban environments likely comes from complex interactions among multiple anthropogenic influences. A recent study (Dominoni et al., 2020) suggests that understanding these interactions can help reveal the presence of ‘sensory danger zones’ for organisms and in turn foster strategic interventions for conservation.

Sexual selection research can be at the forefront of the development of urban evolution, as the multiple biological and landscape effects of urbanization provide unique opportunities for field testing hypotheses regarding multimodal signaling, trait compensation, the role of sexual selection during adaptation, and the evolution of signal honesty. Urban studies can provide natural experiments to test big, unresolved questions in sexual communication and evolution research. For example, urban studies have so far shown mixed evidence for trait compensation, when the relative importance of different sexual signals shift in response to altered environments (de Jong et al., 2018; Rios‐Chelen et al., 2015; Troianowski et al., 2014). Additional work in urban systems can help us understand whether the particular attributes of a study system (e.g., taxonomy, mating system characteristics, and ecological conditions) influence whether or not trait compensation occurs, and if it can help populations weather urban‐caused environmental change. Furthermore, understanding how sexual communication responds to urbanization can complement research on urban speciation (Halfwerk, 2021; Thompson et al., 2018) to help deepen our understanding of which environmental factors are the most potent drivers of diversification and how quickly changes can occur. Urban research may help shed light on the relative roles of natural and sexual selection during population differentiation, and possibly speciation.

### 7.2 Helping cities become refugia

Successful reproduction is crucial for population viability, and a more comprehensive understanding of how sexual communication responds to urbanization will yield more effective conservation insights. An important step for better understanding how to design cities that harbor biodiversity is to conduct more research that is able to disentangle causal agents driving trait differentiation (McDonnell & Hahs, 2015). Urbanization encompasses a complex suite of environmental changes like increased noise and light pollution, and shifts in community structure and resource quality. Clarifying the mechanisms underlying signaling differences between populations in rural and urban environments will be critical for developing conservation recommendations for how to mitigate the negative effects of urbanization. Given the vast heterogeneity in urban design features (e.g., variation in noise and light pollution across urban landscapes) there should be opportunities for observational studies that simultaneously examine how multiple aspects of the environment are associated with traits related to sexual communication. Studies like Narango and Rodewald (2016) serve as a model for teasing apart the relative importance of different ecological agents in promoting diversification and therefore provide insight into urban design changes that will create the biggest impact on wildlife. Understanding the aspects of urbanization that are the strongest drivers of trait changes will help urban designers and conservation managers better focus their efforts on mitigation.

Many of the changes in sexual communication that occur in response to urbanization are due to phenotypic plasticity. This is encouraging from a mitigation standpoint because in many cases, populations should be resilient and responsive to changes in city structures designed to interfere less with animal communication. For example, Derryberry et al. (2020) showed that bird singing behaviors can quickly respond to changes in urban noise pollution. During the COVID‐19 shutdown in San Francisco, CA, noise pollution from traffic plummeted to levels comparable to the 1950s. In this temporarily quieter soundscape, birds sang higher quality songs. Reducing noise pollution has a fairly straightforward solution that is demonstrated to positively affect sexual communication—reduce the number of vehicles on the roads. Cities may accomplish this by investing in infrastructure for residents to safely walk and ride bikes for transportation, or by scaling up public transportation options. An added bonus of greater reliance on walking and bicycles is less carbon emissions from gasoline use.

Fireflies may serve as a useful model for understanding how to better design urban areas to accommodate wildlife. Many firefly species are vulnerable to extinction, and masking of sexual signals by artificial light at night was highlighted as a major threat (Lewis et al., 2020). Due to public safety concerns and general livability of cities, prohibiting ALAN is generally not a feasible solution, though it is the most biologically effective at minimizing the negative effects of ALAN (Gaston et al., 2012). However, recent work has demonstrated that fireflies are less sensitive to longer wavelengths of light (Owens et al., 2018). Therefore, one implementable solution that emerges from this research on fireflies is to replace low‐frequency streetlights with ones that emit longer frequencies. The island of Hawaii (“Big Island”), home to the W. M. Keck Observatory telescopes, has successfully overhauled their streetlight infrastructure to help mitigate light pollution. The new lights are more directional and use LED so emit yellow‐toned, rather than broad‐spectrum light that does not interfere with the astronomy research occurring on the island. There may be other technical advances for light sources that can strike a balance between human needs in urban areas and biodiversity conservation (Gaston et al., 2012). Research on the impacts of urbanization on sexual communication and its consequences will be crucial for clarifying how to create urban design and policies that support rather than degrade diverse biological communities.

## CONFLICT OF INTEREST

The authors have no conflict of interest to declare.

## AUTHOR CONTRIBUTIONS

**Justa L. Heinen‐Kay:** Conceptualization (lead); writing–review and editing (lead). **Adam D. Kay:** Conceptualization (supporting); writing–review and editing (supporting). **Marlene Zuk:** Conceptualization (supporting); writing–review and editing (supporting).

## Data Availability

The manuscript contains no data.
